# From Farm to Fork: Crickets as Alternative Source of Protein, Minerals, and Vitamins

**DOI:** 10.3389/fnut.2021.704002

**Published:** 2021-08-10

**Authors:** Dorothy K. Murugu, Arnold N. Onyango, Alex K. Ndiritu, Isaac M. Osuga, Cheseto Xavier, Dorothy Nakimbugwe, Chrysantus M. Tanga

**Affiliations:** ^1^International Centre of Insect Physiology and Ecology (icipe), Nairobi, Kenya; ^2^Department of Food Science and Technology, Jomo Kenyatta University of Agriculture and Technology, Nairobi, Kenya; ^3^Department of Environmental Health, University of Kabianga, Kericho, Kenya; ^4^Department of Animal Science, Jomo Kenyatta University of Agriculture and Technology, Nairobi, Kenya; ^5^Department of Food Technology and Nutrition, School of Food Technology, Nutrition and Bioengineering, Makerere University, Kampala, Uganda

**Keywords:** edible crickets, *Scapsipedus icipe*, *Gryllus bimaculatus*, nutrient quality, recommended nutrient intake, human food

## Abstract

Globally, there is growing interest to integrate cricket-based ingredients (flour) into food products to combat food and nutrition insecurity. However, there is lack of information on in-depth nutrient profile of the two cricket species (*Scapsipedus icipe* and *Gryllus bimaculatus*), which are the most widely consumed in Africa. Here we determined the nutrient composition of two cricket species and compared them with published records of key animal and plant sources. Our results revealed that the crude protein contents of *S. icipe* and *G. bimaculatus* were similar (56.8 and 56.9%, respectively) and comparable to those of animal protein sources. Both cricket species had balanced amino acid profiles that are superior to that of animal and plant sources, except for histidine and cysteine. The protein digestibility of *S. icipe* and *G. bimaculatus* ranged between 80 and 88%, which is comparable to that of common plant foods but slightly lower than that of animal proteins. The iron, Zinc, and potassium contents were considerably higher in both cricket species compared to that of plant and animal sources. The calcium contents of both crickets (*S. icipe* and *G. bimaculatus*) was superior to that of plant and animal origin except for kidney beans and eggs, respectively. Riboflavin, thiamine, and folic acid concentrations of *S. icipe* and *G. bimaculatus* were superior to that of the conventional sources. Vitamin A levels were significantly higher in *S. icipe* compared to *G. bimaculatus*. This implies that *S. icipe* and *G. bimaculatus* can adequately contribute to our daily required nutrient intake. Thus, integrating cricket flours into ready-to-eat food products would address some of the most pressing nutritional deficiency challenges that many developing countries have to grapple with, particularly high risk to serious health problems such as anemia, poor pregnancy outcomes, hypertension, increased risk of morbidity and mortality, stunted growth and impaired physical and cognitive development. We conclude that edible crickets present unique opportunities for improving food and nutritional insecurity status of both resource-poor and Western populations.

## Introduction

The world's population is expected to reach 9.2 billion in 2050, with most increases occurring in less developed regions (1, 2). Therefore, the worldwide demand for food and meat is likely to increase by 50 and 85%, respectively (3). The vast majority of the hungry people−827 million—live in developing regions and Africa remains the region with the highest prevalence of undernourishment with sub-Saharan Africa (SSA) accounting for a prevalence of 24.8% (4). Among the undernourished populations, over 2 billion people are affected by micronutrient deficiencies globally (5). In terms of global public health significance, iron, zinc, and vitamins are the most important micronutrients (6, 7), which play key roles in preventing malnutrition and early stunting (7). The deficiency of these nutrients is prevalent in areas where there is high cereal and low animal products consumption (8), especially resource-poor areas where the situation is exacerbated by infectious diseases (5). Besides the traditional nutrient deficiency diseases, there is a rising prevalence of non-communicable diseases in both developing and developed countries. Thus, deficiencies of these important nutrients have negative effects on many physiological systems and health, leading to high economic burden for many countries with increasing risk of morbidity and impaired physical and cognitive development (9) as well as poor pregnancy outcome (10, 11).

In SSA, the situation is projected to worsen over the next decades unless drastic measures are taken to reverse food insecurity (5). Efforts to tackle this problem have largely focused on enhancing crop and livestock productivity, which are becoming unsustainable due to dwindling arable land, and water scarcity caused by climate change. Livestock and crop production has been blamed for its significant contribution to negative environmental impact, particularly responsible for huge greenhouse gas emissions (high ecological blueprints), which could possibly contribute to global warming and severe environmental degradation (7).

Thus, there is an urgent need for alternative nutrient sources, and edible insects are promising and potential choice (12). Globally, over 2,000 insect species are consumed by approximately 2 billion people as delicacy with well-documented evidence that they can significantly provide the amount of daily nutrient requirements for human nutrition, especially for children and women of reproductive age (8), thus a potential solution to malnutrition and growing food insecurity globally (13, 14). This explains why Food and Agriculture Organization of the United Nations (FAO) and other stakeholders have embarked on the promotion of edible insects for food (15, 16). Globally, insects from the order Orthoptera such as crickets are one of the most widely farmed and nutrient-rich edible insect group, generating income for women and youths, who represents more than 60% of all the medium- or large-scale enterprises (12). In many cases the nutrient profile of these cricket species is comparable to conventional livestock meat and crops in terms of quality and quantity (17). It has been postulated that crickets generally contain high-quality nutrients, which are easily digestible and more bio-available than those available from plant and animal food sources (18).

Thus, cricket consumption could be immediate solutions to many of the nutrient deficiency issues and can be both inexpensive and effective option. According to several researchers, multisectoral approaches that are tailored to the sustainable utilization of local materials such as insects and that consider the specific conditions of the people would be easily favored by policy makers in view of their sustainability. This explains why the FAO and WHO have recommended dietary diversification, food fortification and supplementation to simultaneously control infections and prevention of other nutritional deficiencies (19). So far, little attention has been paid to crickets as source of essential nutrients in human diets. This study seeks to provide a comprehensive dietary benefits of the newly described cricket *Scapsipedus icipe* Hugel & Tanga (20) and the two-spotted cricket *Gryllus bimaculatus* De Geer (21) in Africa, which are most widely consumed in terms of their richness in minerals, protein, fat, amino acids, and vitamins. Next, we discussed bioavailability of amino acids, vitamins, and minerals in relation to Recommended Dietary Allowance (RDA) published for animal and plant-based sources and outline future directions for research.

## Materials and Methods

### Sample Collection and Preparation

Adult crickets were obtained from subsistence and commercial farms in Western part of Kenya. Samples of each species were collected separately in 1 kg Ziplock bags, frozen alive at −20°C in a Hisense chest freezer (model number: 1159Q61, China) and transported frozen in cooler boxes lined with ice packs to the International Centre of Insect Physiology and Ecology (*icipe*) laboratories for analysis. The raw cricket samples were thawed and blended into a paste using a domestic blender (Signature SG-201, China). The insect paste was subjected to various chemical analysis as described below.

### Proximate Analysis

Proximate components were determined using Association of Official Analytical Chemists (22) methods. The moisture content was determined by oven drying method at 135°C for 2 h (Method No. 930.15) (22). Ash content was determined by ignition of samples at 550°C in a muffle furnace until the weight remained constant (Method No. 930.05) (22). The crude fat content was determined by diethyl ether extraction in a fat extraction unit (SER 148/6; Velp Scientific, Usmate, Italy) following the Randall technique (Method No. 2003.05) (22). The crude protein content of the cricket powder was determined following the Kjeldahl method and the values multiplied by a conversion factor of 6.25 (Method No. 2001.11) (22). Crude fiber was determined by loss of ignition on weight of residue after hydrolysis with acid and alkali solutions (Method number 978.10) (23).

### Determination of Amino Acid Composition

The amino acid composition was determined as previously described by Cheseto et al. (24). The cricket powder (100 mg) was transferred into a 5 mL micro-reaction vial into which 2 mL of 6N HCl was added and closed after careful introduction of nitrogen gas. The sample was hydrolyzed for 24 h at 110°C. After the hydrolysis, the mixtures were evaporated to dryness under vacuum. The hydrolysates were reconstituted in 1 mL 0.01% formic acid/acetonitrile (95: 5), vortexed for 30 s, sonicated for 30 min, and then centrifuged at 14,000 rpm and the supernatant analyzed by LC-MS. The same procedure was performed to determine basic amino acid by substituting 6N HCl with 6N NaOH.

The chromatographic separation was achieved on an Agilent system 1100 series (MA, USA) using ZORBAX SB-C18, 4.6 × 250 mm, 3.5 μm column, operated at 40°C. Mobile phases used were made up water (A) and 0.01% formic acid in acetonitrile (B). The following gradient was used: 0–8 min, 10% B; 8–14 min, 10–100% B; 14–19 min, 100% B; 19–21 min, 100-10% B; 21–25 min, 10% B. The flow rate was held constant at 0.5 ml min^−1^ and the injection volume was 3 μL. The LC was interfaced to a quadruple mass spectrometer. The mass spectrometer was operated on ESI-positive mode at a mass range of m/z 50–600 at 70 eV cone voltage.

Serial dilutions of the authentic standard containing 18 amino acids (1–105 μg/μl, Sigma–Aldrich, St. Louis, MO, USA) was also similarly analyzed by LC-MS to generate linear calibration curves (peak area vs. concentration) used for external quantification. Amino acid analysis was repeated three times using different batch of samples.

### Determination of *in-vitro* Protein Digestibility

Protein digestibility in the insect samples was analyzed by the method described by Mertz et al. (25). Initial protein content of the samples was determined using micro-Kjeldahl nitrogen determination method. This was followed by pepsin digestion, where 0.2 g of the sample was weighed into 50 mL centrifuge tubes. Then 20 mL buffered pepsin was added and mixed. Similarly, a blank was prepared but without a sample. The tubes were placed in a water bath at 37°C for 2 h with gentle shaking after every 20 min. The tubes were then centrifuged at 6,000 rpm for 15 min at 4-degree celcius. The supernatant was discarded, and 10 mL of buffer solution added, then shaking and centrifugation was done again. The supernatant was discarded, and the residue filtered using a Whatman filter paper No. 4. The filter paper was rolled and inserted into a Kjeldahl flask and dried for 15 min in the oven at 100 degrees celcius. Ten (10) mL of Concentrated sulphuric acid, 1 g potassium sulfate and 1 mL of 10% copper sulfate solution were added to the Kjeldahl flask containing the dried filter paper and sample. Then digestion, distillation and titration were done according to the micro-Kjeldahl nitrogen determination.

Protein digestibility (%) = (A - B)/A

Where A = % protein content in the sample before pepsin digestion

B = % protein in the sample after pepsin digestion.

### Determination of Mineral Composition

The cricket powder was ashed and digested in 6N HCl and the content of the various minerals (Iron, Zinc, Calcium, magnesium, sodium, potassium, manganese, copper and cobalt) determined using atomic absorption spectrometry (AAS) (Shimadzu, AA-6300, Tokyo, Japan) according to AOAC methods (23).

### Determination of Vitamin Content

The vitamin content of cricket samples was determined for selected fat-soluble (A, E, pro D) and water-soluble (B1, B2, B3, B6, B9) vitamins. We adopted Cheseto et al. (26) and Jermacz et al. (27) methods for the analysis of fat-soluble vitamins, briefly, each cricket sample (300 mg), was transferred into a 10 mL glass vial containing a mixture of hexane, methanol and distilled deionized water (2:1:2, 5 mL), vortexed for 30 s, sonicated for 30 min and centrifuged at 14,000 rpm for 5 min. The supernatant was dried over anhydrous Na_2_SO_4_, evaporated to dryness under a gentle stream of N_2(g)_ before derivatizing any residual fatty acids to fatty acid methyl esters to limit the matrix interference following the protocol described elsewhere (28), analyzed (1.0 μL) by GC-MS on a 7890 A gas chromatograph linked to a 5,975 C mass selective detector (Agilent Technologies Inc., Santa Clara, CA, USA). The GC was fitted with a (5%-phenyl)-methylpolysiloxane (HP5 MS) low bleed capillary column (30 m × 0.25 mm i.d., 0.25 μm; J&W, Folsom, CA, USA). Helium at a flow rate of 1.25 mL min^−1^ served as the carrier gas. The oven temperature was programmed from 35°C to 285°C, with the initial temperature maintained for 5 min, with a rise at 10°C min^−1^ to 280°C, and then held at this temperature for 20.4 min. The mass selective detector was maintained at ion source temperature of 230°C and a quadrupole temperature of 180°C. Electron impact (EI) mass spectra were obtained at the acceleration energy of 70 eV. Fragment ions were analyzed over 40–550 m/z mass range in the full scan mode. The filament delay time was set at 3.3 min. Serial dilutions of the authentic standard α-tocopherol (≥ 95.5% purity) (0.1–100 ng/μL, Sigma- Aldrich, St. Louis, MO) was analyzed by GC-MS in full scan mode to generate a linear calibration curves (peak area vs. concentration) which gave coefficient of determinations *R*^2^ = 0.9999. The regression equation was used for the external quantification of the different selected fat- soluble vitamins (Retinol, γ-tocopherol, α-tocopherol, and Pro vitamin D).

These compounds were identified by comparison of mass spectral data and retention times with those of authentic standards and reference spectra published by library–MS databases: National Institute of Standards and Technology (NIST) 05, 08, and 11. The samples were analyzed in triplicate, with each replicate collected from a different batch of respective samples.

Determination of water–soluble vitamins was carried out according to previously described method (28, 29). Briefly, 100 mg of each cricket sample was transferred in to a 50 mL falcon tube containing 25 mL distilled deionized waster (25 mL), vortexed for 20 s, sonicated for 15 min and the mixture filtered through 0.2 μm filters into Ultra Performance Liquid Chromatography (UPLC) vials and analyzed by Shimadzu UPLC-DAD. The chromatographic analysis was performed on (LC-30AC with Nexera column oven CTO-30A, Shimadzu, Tokyo, Japan) fitted with a Phenomenex C_18_ Column Synergi 100 mm × 3.00 mm, 2.6 μm polar (Phenomenex, Torrance, CA, USA) at 30°C. The mobile phase consisted of two phases, A: 25 mM phosphate buffer. B: 7:3 v/v Acetonitrile-Mobile phase A. Total run time was 12 min with a flow rate of 0.4 mL/min. Stock solutions of 1.0 mg/ml were prepared by dissolving the individual water-soluble vitamin standards in distilled water except for Vitamin B_2_ in (5 mM potassium hydroxide) and Vitamin B_9_ in (20 mM potassium hydrogen carbonate). Serial dilutions of the stock solution (2–15 μg/mL) for the 5-water-soluble vitamins were also analyzed by UPLC-DAD giving R^2^ of 0.996 or greater. These regression equations were used for the external quantification of the different water-soluble vitamins. All determinations were carried out in triplicates from different batch of respective samples.

### Data Analysis

Data were checked for normality using the Shapiro-Wilk test. To determine the differences in nutritional value of the two cricket species, unpaired *t*-test was used for normally distributed data with equal variances while Welch's *t*-test was used to analyse data that did not fulfill the two assumptions. All the statistical analyses were conducted using R software version 3.6.0 (30).

## Results and Discussion

### Proximate Composition *Scapsipedus icipe* and *Gryllus bimaculatus*

**Figure 1:**
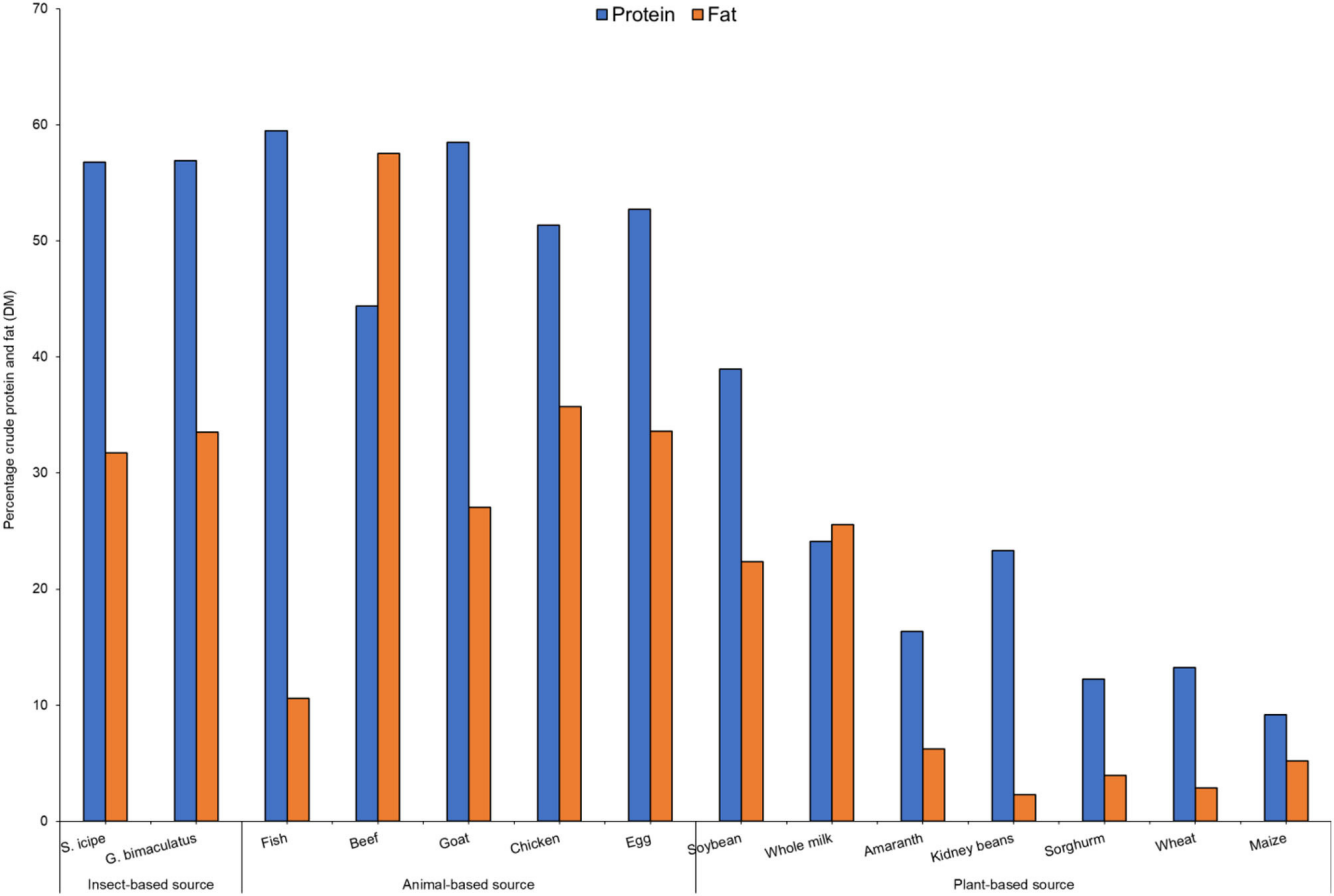
Comparative analysis of percentage crude protein and fat content [on dry matter basis—DM] of crickets (*Scapsipedus icipe* and *Gryllus bimaculatus*), and selected plants and animal sources. Data for plant and animal sources was extracted from FAO/GoK (31).

The proximate composition (on dry matter basis-DM) of *S. icipe* and *G. bimaculatus* are presented in Figure 1. The crude protein (CP) content of *S. icipe* and *G. bimaculatus* was comparable to that reported previously for field cricket (32, 33). However, the CP values in the present is relative lower compared to reported for *Acheta domesticus* Linnaeus (73.63%) (34). The variation can be attributed to inter-species differences as well as the type of substrates fed to cricket during rearing (35). The crude fat recorded for *Gryllus bimaculatus* was significantly (*P* = 0.0005) higher compared to that *S. icipe*. These results are consistent to that reported for *A. domesticus* (32.6 %) (36) but contrary to that documented in other study for the same species (18.55–22.80%) (37). Based on the crude fat content, it implies that the consumption of about 200 g of crickets would potentially contribute the daily requirement of energy from fat for human nutrition, which ranges between 10 and 30% (38).

The values of crude ash reported in this study (Table 1) were comparable to that reported for *A. domesticus* (3.57%) (39). This aligns with results reported for adult cricket under rearing conditions (3.57–5.10% crude ash) in the United States of America (USA) (37). The crude fiber content of *S. icipe* and *Gryllus bimaculatus* are comparable to the values (5.95 and 8.7%) reported by Finke (40) and Moreki et al. (32) for crickets, respectively. Previous studies have demonstrated that the amount of crude fiber in an insect is a direct reflection of the chitin on the basis of the chemical structure (39, 40). In literature it is reported people of the African origin have high activity of chitinase enzyme, thus this opens new opportunities for the promotion and commercialization of edible insects chitin (41). Recently, chitin and chitosan have attracted considerable attention due to their biological activities (antifungal, antibacterial, antitumor, immunoadjuvant, antithrombogenic, anti-cholesteremic agent) and bio-adhesivity (42). Thus, chitin is widely used as absorption promoters and hydrating agents, as well as for film production and wound healing (43). The immunity-enhancing effects, promotion of beneficial bacterial growth and inhibition of pathogenic microorganisms have been reported, thus demonstrating clear health benefits after consumption (44–48). The application of chitin/chitosan for extension of shelf life of various foods from agriculture, poultry, and seafood origin by inhibiting microbial growth have been documented (49).

**Table 1:** Crude ash, crude fiber, crude protein, crude fat, and energy levels of *Scapsipedus icipe* and *Gryllus bimaculatus* on dry matter basis (mean ± SE).

**Cricket species**	**Crude ash**	**Crude fiber**	**Crude protein**	**Crude fat**	**Energy (Kcal/10 0g)**
	**Percentage (%)**				
*Scapsipedus icipe*	5.25 ± 0.02^a^	5.71 ± 1.39^b^	50.19 ± 2.06^a^	35.70 ± 2.57^b^	512.66 ±1.72^b^
*Gryllus bimaculatus*	5.41± 0.06^a^	8.39 ± 2.28^a^	58.19 ± 3.67^a^	46.04 ± 2.90^a^	529.18 ± 3.17^a^
*t*-test value	−0.36	2.72	1.89	2.67	4.54
df	27.9	14	22.0	27.6	3.1
*P*-value	0.718	0.017	0.071	0.013	0.019

*Means with different superscript letters in each column are significantly different at p < 0.05*.

The protein and fat content of the cricket species studied was comparable to that of common animal foods and higher than most plant sources (Figure 1). These results imply that 100 g of either cricket species consumed per day would provide at least twice as much protein as any of the common plant sources. Studies carried out in South Korea found that protein and fat content of five insect species including *G. bimaculatus* surpassed that of conventional livestock sources (50), which is slightly different from the current results. Therefore, consumption of *S. icipe* and *G. bimaculatus* could provide the much-needed fat and protein in communities where access to traditional animal sources are limited (51).

**Figure 2:**
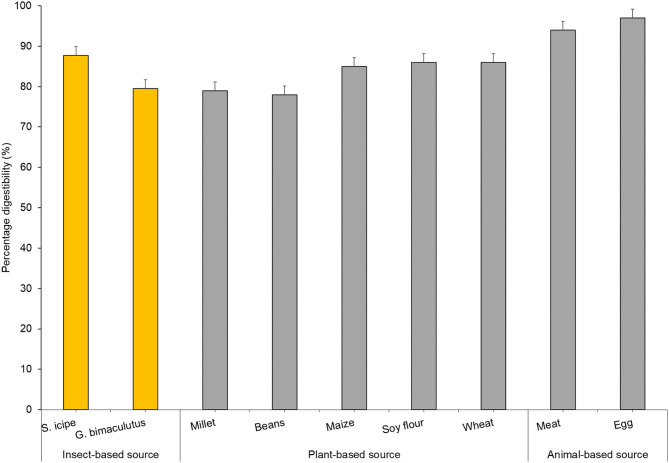
Comparative analyses of *in vitro* protein digestibility of crickets (*Scapsipedus icipe* and *Gryllus bimaculatus*), plants and animal food sources. Data for plant and animal sources was extracted from FAO/WHO/UNU (56).

In general, edible insects in the order Orthoptera have been reported to contain significantly higher amounts of crude proteins compared to other insects (37, 52). However, quantifying bioavailable insect protein has been of great concern given that crude protein analysis using Kjeldahl method often includes nitrogen embedded in the exoskeleton of these insects which largely comprises the polysaccharide chitin, a phenomenon that tends to overestimate digestible protein of edible insects (52–54). *In vitro* protein digestibility has been shown as a reliable predictor of protein bioavailability *in vivo* based on Protein Efficiency Ratio (PER) and Net Protein Ratio (NPR) (29, 55). In this study, there was significant difference in digestibility of protein from both species were observed to be different (Figure 2). The values for *S. icipe* (87.8%) and *Gryllus bimaculatus* (79.5%) are within the range reported for other insects (76–96%) by Kourimská and Adámková (57). Other studies have also reported significantly higher protein digestibility values for the cricket species *Gryllus assimilis* (Fabricius) (73%) when compared to that of grasshopper, moth caterpillar and termite. The protein digestibility corrected for amino acid score of the newly described cricket species in Kenya (*S. icipe*) with 87.77%, is similar to that of egg (97%) and beef (98%) (58). Insect protein digestibility has been considered higher than most plant proteins in previous studies (59).

### Amino Acid Composition

**Figure 3:**
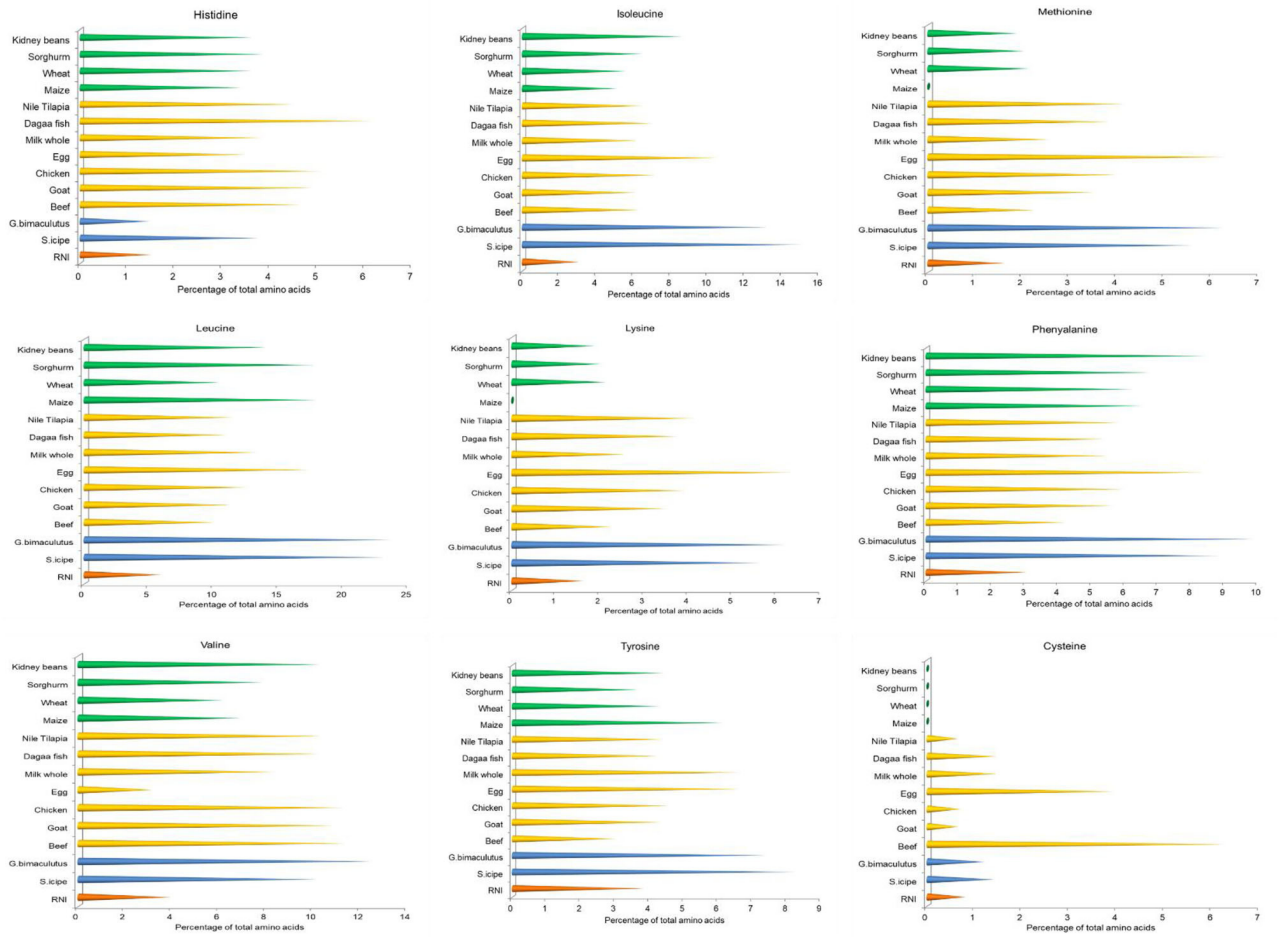
Comparison of selected essential amino acids content of crickets (*Scapsipedus icipe* and *Gryllus bimaculatus*), animal and plant-based sources. Amino acid data for plant and animal sources was extracted from FAO/GoK (31), while that of the recommended nutrient intake (RNI) was obtained from FAO/WHO/UNU (56).

Both *S. icipe* and *Gryllus bimaculatus* were found to have seven essential amino acids as shown in Figure 3. Five of these essential amino acids content varied significantly between *S. icipe* and *G. bimaculatus*. Non-essential amino acids detected *S. icipe* and *G. bimaculatus* are presented in Table 2. Leucine was the most abundant amino acid in *S. icipe* and *Gryllus bimaculatus*. The highest amount of glutamine, glutamic acid, proline, valine, methionine, leucine, and phenylalanine were recorded for *G. bimaculatus*. Histidine and lysine levels were more abundant in *S. icipe* than in *Gryllus bimaculatus*. No significant difference was observed for arginine (*P* = 0.0584), lysine (*P* = 0.2742), tyrosine (*P* = 0.7285), and isoleucine (*P* = 0.9412) between *S. icipe* and *G. bimaculatus*. The high levels of isoleucine and leucine recorded for *icipe* and *G. bimaculatus* are similar to that reported for other edible insects (60). The methionine content in *S. icipe* and *G. bimaculatus* were comparable to that reported for eggs, which is well-known as an excellent source of methionine. Methionine and lysine are the most limiting amino acid in legume and legume products such as bean, peas, lentils, chickpeas, and soybean as well as cereals, which is in line to that reported in other studies (61). Methionine is a classical sulfur amino acid, a limiting amino acid in plant proteins of legume origin and aids availability of cysteine which is a metabolic product of methionine catabolism. The values of methionine, leucine and phenylalanine recorded for *Gryllus bimaculatus* are similar to that reported for *A. domesticus* by Ramos-Elorduy et al. (33). Similarly, methionine and phenylalanine of the field cricket *Gryllus testaceus* Walker are comparable to the results observed in the present study, although, histidine, lysine, glutamic acid, proline, valine, tyrosine were higher except for much lower values for isoleucine and leucine (62). According to a report by FAO/WHO, daily amino acid requirements for human nutrition ranged between 0.010 and 0.039 mg/g (63), thus lower than the value reported for *S. icipe* and *G. bimaculatus* (31, 56, 64). This implies that both cricket species could be an excellent alternative and sustainable source of alternative amino acids than current animal and plant-based sources. Lysine deficient diets are common in African countries, where maize is a staple food, thus supplementing these diets with edible cricket protein would be a sustainable step toward dietary diversification (50) and ensuring nutritional security. However, the protein quality of the edible insects can further be improved with the removal of chitin which binds some amino acids (37).

**Table 2:** Amino acid profile (μg/100 mg) of *Scapsipedus icipe* and *Gryllus bimaculatus* on dry matter basis (mean ± SE).

**Amino acid**	**Cricket species**		***P*-value**	***t*-test value**	**df**
	***Scapsipedus icipe***	***Gryllus bimaculatus***			
Histidine	10.80 ± 0.87^a^	4.83 ± 6.24^b^	0.016	−2.93	9.37
Arginine	13.59 ± 1.06^b^	14.0 ± 1.96^a^	0.038	2.25	18
Lysine	18.21 ± 3.85^a^	15.64 ± 6.74^a^	0.442	−0.79	14.5
Glutamine	Absent	1.75 ± 3.73	–	–	–
Glutamic acid	9.58 ± 0.31^b^	11.34 ± 0.55^a^	<0.001	9.17	13.4
Proline	19.21 ± 6.10^b^	30.47 ± 6.29^a^	0.001	3.85	17.9
Valine	29.14 ±7.84^b^	41.38 ± 8.15^a^	0.007	3.02	17.8
Methionine	16.12 ± 3.87^b^	20.67 ± 3.62^a^	0.034	2.28	18.0
Tyrosine	23.78 ± 5.81^a^	24.49 ± 2.97^a^	0.755	−0.32	14.0
Isoleusine	43.41 ±10.57^a^	43.86 ± 17.44^a^	0.738	−0.34	14.0
Leucine	66.24 ± 12.53^a^	78.56 ± 10.85^a^	0.080	1.86	18.0
Hydroxyproline	8.83 ± 0.51^b^	9.37 ± 0.17^a^	0.025	2.60	11.0
Phenylalanine	25.37 ± 8.00^b^	32.75 ± 3.26^a^	0.036	2.37	11.4

*Means with different superscript letters in each column are significantly different at p < 0.05*.

Histidine is an indispensable amino acid whose dietary deficiency has been shown to cause deleterious effects on hemoglobin concentrations in humans (65, 66), thus consumption of these cricket species could be a quick fix to such health complications. Amino acids such as Leucine, isoleucine, and valine, which are branched chain amino acids (BCAA) were recorded notably in higher amounts in both crickets than in the animal and plant-based sources. Several studies have documented the potential role of the BCAA in brain function and maintenance of muscle mass during weight loss (67–69). Other studies have cited leucine and histidine as essential in growth of children suggesting that both cricket species can be applied to supplemental diets to back up their protein requirements (70, 71). Leucine has a wide range of metabolic and regulatory influences in the body and has been shown to have a potential role in the treatment of obesity and metabolic syndrome due to its influence on insulin secretion and sensitivity as well as dietary macronutrient disposal (72, 73). Therefore, leucine can play a potential role in the prevention of type II diabetes (71, 73). Further, leucine has been shown to play a critical role in the reversal of adverse influences of high fat diet thus facilitating healthy weight maintenance in humans (72, 74, 75). Both cricket species in the present study were observed to have at least twice the amount of the recommended lysine and methionine for children at different age groups as well as adolescents (65). Lysine and arginine are important factors in the release of growth hormone in young children (76). High quality rich foods have been linked to reduced risk of stunting in young children aged 2–13 years (77, 78) and hence incorporating cricket-based protein in diets of young children and complementary or supplementary food products targeted at this most vulnerable segment of the resource poor communities in developing countries would contribute to easy access of diversified and well-balanced diets.

### Mineral Composition

The importance of edible insects as a source of minerals has been documented in several studies (37, 79, 80). Based on the current study, the iron content of the two edible crickets ranged between 10.70 and 12.33 mg/100 g, which is similar to that reported for *A. domesticus* (11.23 mg/100 g) (81). Thus, the consumption of 100 g of *S. icipe* or *G. bimaculatus* will contribute to at least 54 and 100% of the recommended dietary allowance (RDA) of iron for women of reproductive age and children below 5 years of age, respectively (Table 3). Globally, iron deficiency is the commonest nutritional disorder affecting both children and women of reproductive age (82). For example, the anemia prevalence statistics for developing countries show that one in every two pregnant women and 40% of school going children are affected (82). Anemia which causes 20% of maternal deaths, also leads to irreversible health consequences including pre-term babies, poor physical and cognitive development, and increased risk of morbidity in children (82). It is thus, evident that integrating edible crickets into the regular diets of women and children might have the potential to address the anemia problem (61). Recent studies have also shown that acceptability of common staples that are fortified with edible insect meals is high (13) and should be encouraged.

**Table 3:** Mineral composition of edible cricket species (*Scapsipedus icipe* and *Gryllus bimaculatus*) in milligrams/100 grams on dry matter basis (mean ± SE).

**Cricket species**	**Calcium**	**Potassium**	**Magnesium**	**Sodium**	**Iron**	**Zinc**	**Manganese**	**Copper**	**Cobalt**
	**(mg/100 g)**								
*Scapsipedus icipe*	66.07 ± 1.63^b^	66.32 ± 0.33^a^	35.57 ± 1.95^b^	395.44 ± 41.63^b^	10.70 ± 0.60^a^	19.19 ± 0.64^b^	95.67 ± 17.22^a^	7.93 ± 0.73^a^	5.09 ± 2.64^a^
*Gryllus bimaculatus*	72.70 ± 1.74^a^	39.54 ± 0.92^b^	29.13 ± 0.55^a^	166.50 ± 0.01^a^	12.33 ± 5.36^a^	23.74 ± 1.30^a^	108.49 ± 18.25^a^	8.27 ± 1.50^a^	4.36 ± 1.62^a^
*t*-test value	5.6	−46.6	−6.4	−11.0	0.61	6.3	1.0	0.41	−0.5
df	5.9	3.0	3.5	3.0	3.1	4.4	6.0	4.4	5.0
*P*-value	0.002	<0.001	0.005	0.002	0.587	0.003	0.346	0.704	0.658

*Means with different superscript letters in each column are significantly different at p < 0.05*.

**Figure 4:**
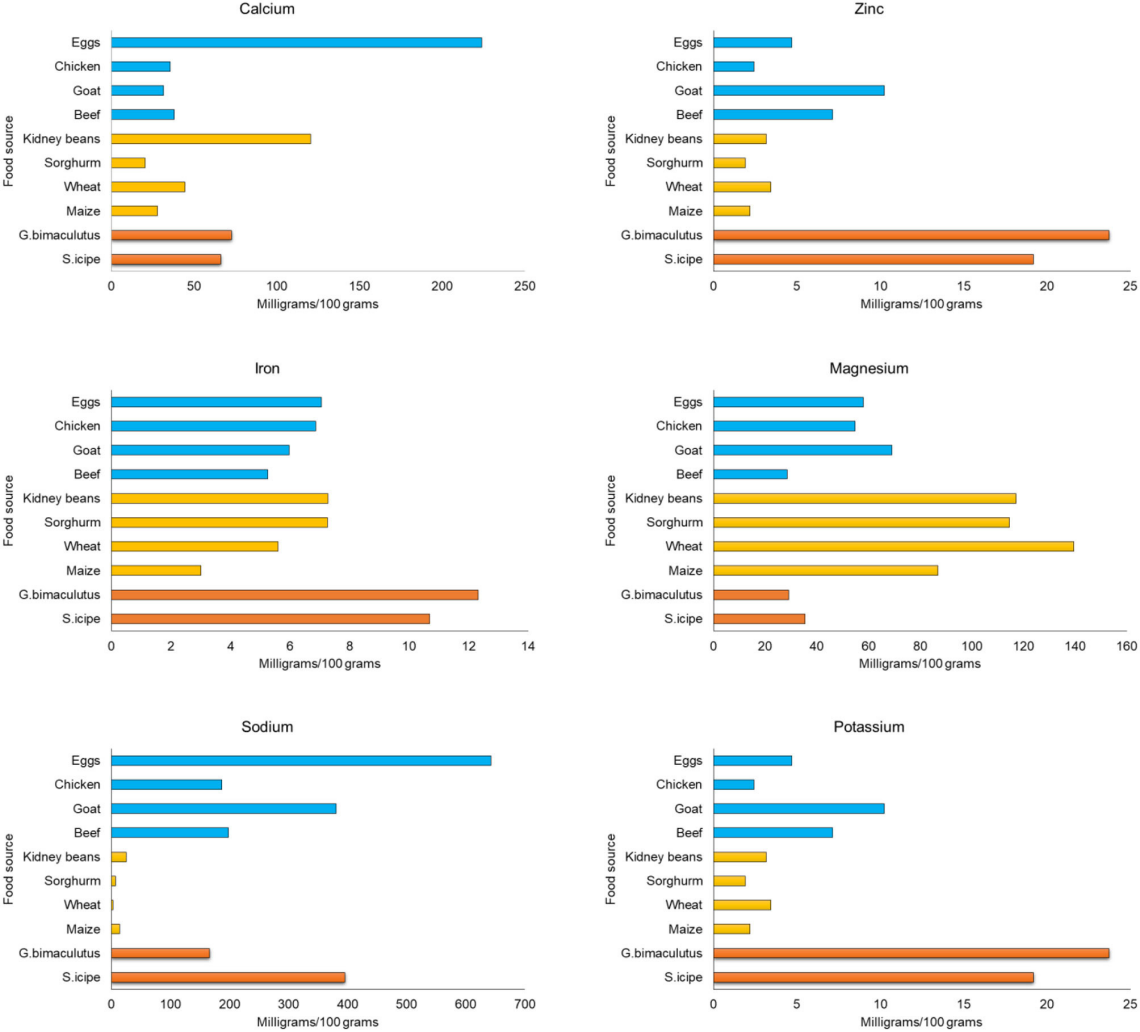
Comparison of mineral content of crickets (*Scapsipedus icipe* and *Gryllus bimaculatus*), animal and plant-based sources. Mineral content data for plant and animal food sources was extracted from FAO/GoK (31), while that of the recommended nutrient intake (RNI) was obtained from FAO/WHO/UNU (56).

Zinc is another mineral of public health importance and the values obtained for *S. icipe* or *G. bimaculatus* in the present study are comparable to that of *A. domesticus* (18.64 mg/100 g) (81) but higher than that reported for *A. domesticus* (13 mg/100 g) (61) and *Gryllodes Sigillatus* (13.9 mg/g) (58). Consumption of 100 g of *S. icipe* or *G. bimaculatus* per day would potentially contribute enough nutrient to meet 100% of the recommended daily allowance for zinc (2–11 mg/100 g) for all age groups. As shown in Figure 4, the two cricket species are clearly superior in zinc content than common animal and plant foods. Thus, they may play a great role in alleviating this most common deficiency, which is associated with stunting and hypertension (83).

Calcium content was higher in *Gryllus bimaculatus* (72.70%) than in *S. icipe* (66.07%). Values obtained in this study are higher than those recorded by Rumpold and Schlüter (36), Mousavi et al. (84) for *A. domesticus*. However, the calcium contents in this study are lower than 130 mg/100 g reported by Rumpold and Schlüter (36) for the edible cricket *A. domesticus*. This wide variation observed between the various studies can be attributed to the choice of diet fed to the crickets (35). It is anticipated that 100 g of either *S. icipe* and *G. bimaculatus* would contribute an estimated 16.5–18% of RDA for children (Table 3). Thus, these edible cricket species could potentially contribute significantly in alleviating calcium deficiencies in many highly vulnerable communities in sub-Saharan African countries, if incorporated in their diet regularly.

Magnesium content was higher in *S. icipe* (35.57 mg/100 g) than in *Gryllus bimaculatus* (29.13 mg/100 g) (Table 3). These values are close to 33.7% and higher than 22.6% previously reported for adult house cricket (*A. domesticus*) (39, 85). Therefore, intake of either *S. icipe* or *G. bimaculatus* food products could potentially contribute to ~50% of RDA of magnesium requirements for young children. The role played by magnesium in the body as a cofactor of enzymes involved in metabolism, synthesis of protein, RNA and DNA and the maintenance of electrical potential of nervous tissues and cell membranes has been well-documented (82).

The potassium content of *S. icipe* or *G. bimaculatus* ranged between 39.54 and 66.32 mg/100 g (Table 4), which is higher than value reported for other studies (37.4 mg/100g) (86). The potassium content of *S. icipe* was close to 74.6 mg/100 g, which is consistent to that reported for the giant African cricket (87). However, other studies have reported higher potassium content in cricket compared to values obtained in this study (39). The variation in potassium content could be attributed to differences in diets and age, as reported in crickets such as *Brachytrypes membranaceus* L. (88). No significant variation of copper content was observed between *S. icipe* and *G. bimaculatus* but the values recorded are higher than those reported for adult *A. domesticus* (0.62–0.85 mg/100 g) (39, 81). The sodium content of *S. icipe* and *G. bimaculatus* varied considerably, though the values were higher in the former than in the later. The sodium content of *Gryllus bimaculatus* was comparable to that reported for adult *A. domesticus* (134 mg/100 g) (39), while that of *S. icipe* was similar to that documented for the same cricket (430 mg/100 g) species (89). A higher potassium: sodium ratio in the two cricket species than common plant and animal foods (Figure 4) implies that they could be suitable source for people with or at risk of hypertension and the metabolic syndrome (90). The manganese content of *S. icipe* and *G. bimaculatus* were comparable. Comparative studies shows that the consumption of these crickets might be capable of contributing adequately to the amount of the RDA of manganese required for children, adolescents, adults, and even lactating mothers (82). However, the values obtained in this study are considerably higher than that reported for other adult crickets which ranged between 2.37 and 3.73 mg/100g (37). Similarly, the manganese content of the two cricket species in this study is significantly higher than the 1.15 mg/100 g reported in cricket species (39). However, further research to confirm this extreme high value of manganese is crucial. Finally, cobalt which is one of the trace minerals and whose main role is being a component of vitamin B_12_ (cyanocobalamin) (91) was recorded in *S. icipe* and *G. bimaculatus*. The value of cobalt in both crickets ranged between 4.36 and 5.09 mg/100 g, which is consistent to that reported in the Snout beetle (4.76 mg/100 g), though higher than those reported for termite (92, 93). This implies that cricket could potentially be a good source of minerals of public health importance such as iron and zinc with values that are superior to those of plant and animal-based food sources (Figure 4).

**Table 4:** Vitamin composition of edible cricket species (*Scapsipedus icipe and Gryllus bimaculatus*) on dry matter basis (mean ± SE).

**Cricket species**	**Retinol (IU)/(mcg/100 g)**	**γ-tocopherol**	**α-tocopherol**	**Provitamin D**	**Vitamin B_**1**_**	**Vitamin B_**2**_**	**Vitamin B_**3**_**	**Vitamin B_**6**_**	**Vitamin B_**9**_**
		**(mg/g)**			**(mg/kg)**				
*Scapsipedus icipe*	139.41 ± 19.19^b^/ (41.847 ± 5.757)	0.48 ±0.13^a^	8.79 ± 4.57^a^	0.43 ± 0.20^a^	0.85 ± 0.11^a^	5.40 ± 1.17^a^	Absent	16.06 ± 4.90^a^	4.12 ± 0.99^a^
*Gryllus bimaculatus*	107.24 ± 26.47^a^/ (32.172 ± 7.941)	0.52 ± 0.27^a^	12.55 ± 0.63^a^	0.22 ± 0.27^a^	4.23 ± 0.67^b^	8.90 ± 5.35^a^	10.93 ± 2.67	52.76 ± 8.67^b^	5.14 ± 1.01^b^
*t*-test value	2.95	0.289	1.87	−1.00	−16.30	−1.68	–	−16.8	−2.17
df	16	4.37	3	3	8	8	–	8	16.0
*P*-value	0.009	0.786	0.158	0.391	<0.001	0.131	–	<0.001	0.046

*Means with different superscript letters in each column are significantly different at p < 0.05; mcg, microgram; IU, International unit; NEs, niacin equivalents; Values in brackets represent retinol content in mcg/100 g*.

### Vitamins

There is insufficient data published on vitamin composition of edible insects and particularly on crickets such as *A. domestica* (57). In this study, the vitamin A (retinol) content of *S. icipe* (42 mcg/100 g) and *Gryllus bimaculatus* (32 mcg/100 g) varied considerably (Table 4). These values of vitamin A are relatively higher than what has been reported from adult domestic house cricket *A. domesticus* (24.33 mcg/100 g) (84), *Brachytrypes spp*. (0 mcg/100 g), adult grasshopper *Cytacanthacris aeruginosus* unicolor (1 mcg/100 g), short horned grasshoppers (6.82 mcg/100 g), *Analeptes trifasciata* (12.54 mcg/100 g), *Anaphe infracta* (2.95 mcg/100g), *Anaphe recticulata* (3.40 mcg/100 g), *Anaphe* spp. (2.78 mcg/100 g), *Anaphe venata* (3.12 mcg/100 g), *Cirina forda* (2.99 mcg/100 g), *Apis mellifera* (12.44 mcg/100 g), *Oryctes boas* (8.58 mcg/100 g), and *Rhynchophorus phoenicis* (11.25 mcg/100 mg) (88). Therefore, consumption of about 100 g of *S. icipe* (newly described species in science) can contribute to approximately 22% RDA of Vitamin A (retinol) among young children (Table 4). Children in sub-Sahara Africa and other low-income countries are particularly at risk of vitamin A deficiency (VAD), thus *S. icipe* and *G. bimaculatus* can be excellent promising and sustainable source of this vitamin (82).

The content of γ-tocopherol (*P* = 0.7823), alpha-tocopherol (*P* = 0.1536), and provitamin D observed in *S. icipe* and *G. bimaculatus* did not vary significantly. The values reported in this study for γ- and α-tocopherol were considerably higher than that of vitamin E content (0.072 mg/g) reported in reared cricket species such as *A. domesticus* (37). The high level of discrepancy in results of tocopherol concentrations in edible crickets could be attributed to the type of feeding substrate used (94).

The provitamin D concentration of the edible crickets ranged between 0.22 to 0.43 mg/g. However, there was no statistical significance difference (*p* = 0.2611) in provitamin D concentration between the two cricket species. The provitamin D concentration recorded for *S. icipe* and *G. bimaculatus* (0.22–0.43 mg/g) is much higher than 0.0064 mg/g reported for *A. domesticus* (39). This implies that the consumption of *S. icipe* and *G. bimaculatus* could also potentially contribute to the RDA for children, adolescents, adults, and lactating mothers.

However, the concentration of thiamine (vitamin B_1_) in *Gryllus bimaculatus* (4.23 mg/kg) was significantly higher when compared to that of *S. icipe* (0.85 mg/kg) fed on the same diet. The vitamin B_1_ content recorded for *Gryllus bimaculatus* is comparable to that reported for *A. domesticus* (3.6 mg/kg) (53). On the contrary the vitamin B_1_ of the two cricket species studied were considerably lower than 15.2 mg/kg reported for *A. domesticus* reared in Kenya (95). Contrarily, the Thiamine concentrations in *S. icipe* and *G. bimaculatus* are higher comparatively to that reported for other cricket species (0.4 mg/kg) (39). These differences observed could be associated to species difference and variation in rearing diet formulation (96).

For vitamin B_2_ (Riboflavin), no significant differences were observed (*p* = 0.0728) between *S. icipe* and *G. bimaculatus*. However, our values are comparable to that reported for the long-horned grasshopper *Ruspolia differens* (12.8 mg/kg) (97) and crickets (19.1 mg/kg) (98). The vitamin B_2_ content of the two cricket species reported in the present study is higher than what has been documented for other Orthopteran species (0.3–0.8 mg/kg) (99) and other commonly eaten insects in Southwestern Nigeria (0.03–3.24 mg/100 g). The variation in vitamin B_2_ content between literature and the findings of this study could be attributed to differences in species, age and feeding regime (96). Notably, niacin was not detected in *S. icipe* but the value recorded for *G. bimaculatus* (10.93 mg/kg) are lower compared to previous studies on crickets with values ranging between 31.0 and 38.4 mg/kg (39, 53).

The concentrations of vitamin B_6_ (pyridoxine) among the two cricket species differed significantly (*p* < 0.0001) with *Gryllus bimaculatus* recording the highest value (52.76 mg/kg) compared to *S. icipe* (16.06 mg/kg). The vitamin B_6_ concentrations observed in both crickets are lower than values (74.7 mg/kg) reported for other edible crickets (37).

**Figure 5:**
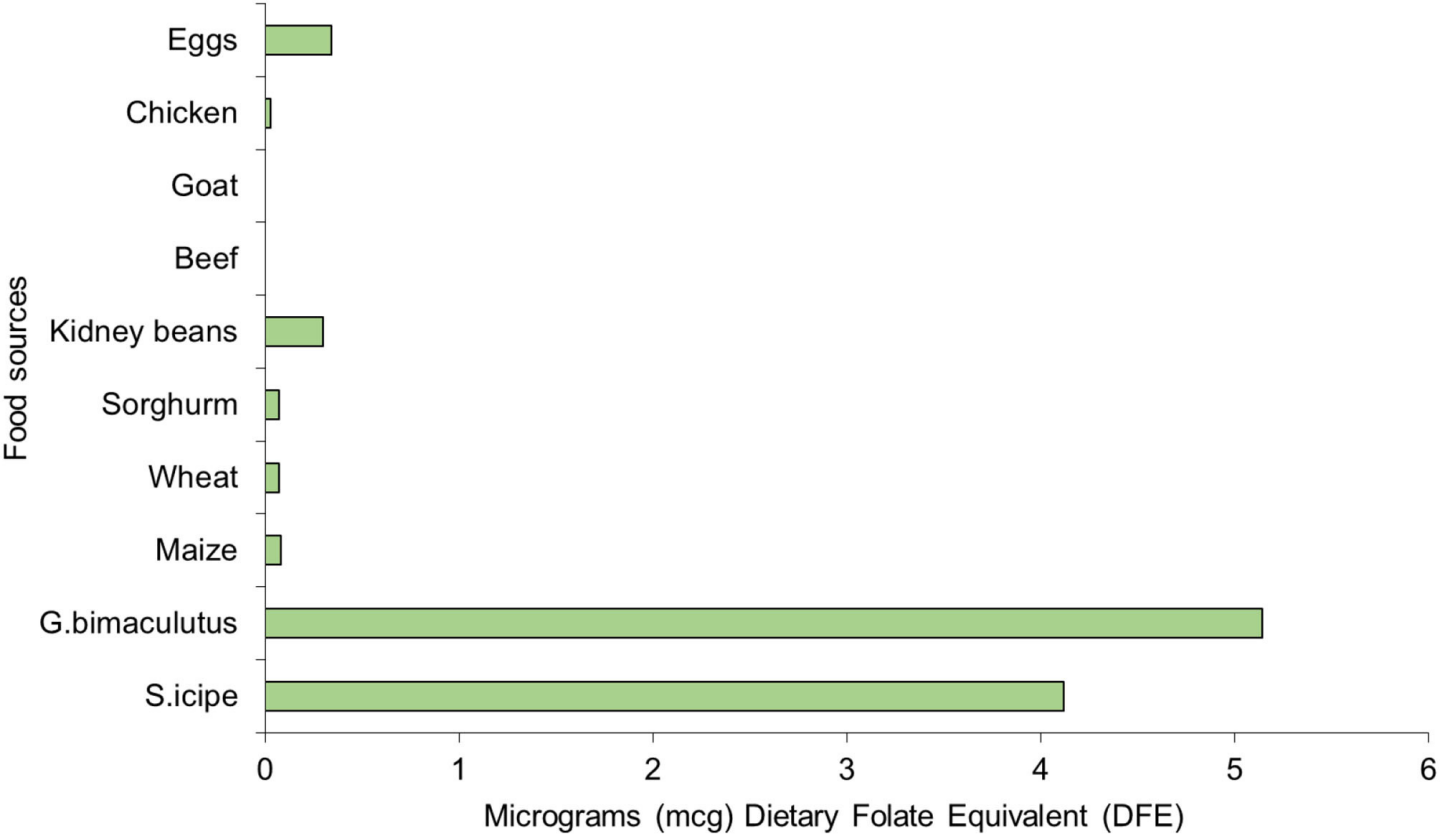
Folic acid content [microgram Dietary Folate Equivalent (mcg DFE)] of *Scapsipedus icipe* and *Gryllus bimaculatus*, animal and plant-based sources. Data for plant and animal food sources was extracted from FAO/GoK (31).

The folic acid content of *S. icipe* (4.12 mg/kg) and *Gryllus bimaculatus* (5.14 mg/kg) were comparable but much higher than values reported for other edible crickets (1.5 mg/kg) in literature (39). Similarly, the folic acid content of the two cricket species is higher than what has been reported in Tenebrio molitor (1.37 mg/kg) (100). These levels of folic acid are also remarkably higher than that in some common foods (Figure 5) of animal and plant origin (Figure 5). Folic acid is a vitamin of public health significance whose deficiency is being addressed through folic acid supplementation and food fortification, especially during pregnancy to reduce cases of birth defects and consequently morbidity and mortality rates among children (100). Thus, *S. icipe* and *Gryllus bimaculatus* could be promising and cheaper alternative to commercial folic acid supplements. Similarly, the riboflavin levels in *S. icipe* and *G. bimaculatus* are significantly higher when compared to that of animal and plant-based food sources. It remarkable to note that *S. icipe* and *Gryllus bimaculatus* can significantly contribute to the recommended nutrient intake (RNI) of many vitamins such as riboflavin, thiamine, folic acids, and niacin, which are very critical for males, females, children, pregnancy, and lactating mothers as shown in Table 4 and Figure 5.

## Conclusion

*Scapsipedus icipe* and *G. bimaculatus* present unique opportunities for improving the nutritional status of resource-poor and affluence populations toward addressing macronutrient and micronutrient malnutrition globally. Our results have demonstrated that the described cricket species can significantly contribute to the daily nutrient requirements of children and adults and children, particularly minerals (iron, zinc, calcium and others), protein, vitamins, and essential amino acids. It is important to note that crickets have featured in human diets around the world for many decades with their consumption widely practiced in parts of Africa, Asia, and Latin America. This partly explains why these insects are receiving increasing attention for their potential to alleviate the projected food and protein demand by the rapidly growing global population. Although, the sustainable utilization of crickets to contribute to our daily nutrient intake has received inadequate research attention globally, our results generated demonstrate the urgent need for their inclusion (whole, in-part, or processed flour) into human diet. This will play an important role toward the achievement of the “zero hunger” sustainable development goal target, given that 98% of the 795 million individuals suffering from hidden hunger live in low- and middle-income countries, where cricket consumption are widely accepted. Thus, there is urgent need for innovative community-based strategies for scaling up the coverage of cricket consumption. However, further studies on the bioavailability of Fe, Zn and folic acids from cricket-derived food products are required to quantify their overall dietary and therapeutic benefits. Also, factors that may interfere with nutrient intake or absorption such as processing, preservation, preparation, and incorporation into other foods needs to be addressed.

## Data Availability Statement

The original contributions presented in the study are included in the article/supplementary material, further inquiries can be directed to the corresponding author/s.

## Author Contributions

DM, AO, AN, IO, CX, DN, and CT: conceptualization, investigation, writing—review, and editing and final approval. DM and CT: data curation and formal analysis. CT: funding acquisition, project administration, and resources. DM, CX, and CT: methodology. AO, IO, and CT: supervision, validation, and visualization. DM, AO, IO, and CT: writing—original draft. All authors contributed to the article and approved the submitted version.

## Author Disclaimer

The views expressed herein do not necessarily reflect the official opinion of the donors.

## Conflict of Interest

The authors declare that the research was conducted in the absence of any commercial or financial relationships that could be construed as a potential conflict of interest.

## Publisher's Note

All claims expressed in this article are solely those of the authors and do not necessarily represent those of their affiliated organizations, or those of the publisher, the editors and the reviewers. Any product that may be evaluated in this article, or claim that may be made by its manufacturer, is not guaranteed or endorsed by the publisher.
